# Aerosols chemical composition, light extinction, and source apportionment near a desert margin city, Yulin, China

**DOI:** 10.7717/peerj.8447

**Published:** 2020-02-14

**Authors:** Yali Lei, Zhenxing Shen, Zhuoyue Tang, Qian Zhang, Jian Sun, Yongjing Ma, Xiaoyan Wu, Yiming Qin, Hongmei Xu, Renjian Zhang

**Affiliations:** 1 Department of Environmental Sciences and Engineering, Xi’an Jiaotong University, Xi’an, Shaanxi, China; 2 School of Environmental & Municipal Engineering, Xi’an University of Architecture and Technology, Xi’an, Shaanxi, China; 3 College of Atmospheric Sciences, Key Laboratory of Arid Climatic Change and Reducing Disaster of Gansu Province, Lanzhou University, Lanzhou, China; 4 State Key Laboratory of Atmospheric Boundary Layer Physics and Atmospheric Chemistry (LAPC), Institute of Atmospheric Physics, Chinese Academy of Sciences, Beijing, China; 5 School of Engineering and Applied Sciences, Harvard University, Cambridge, United States of America; 6 Key Laboratory of Regional Climate-Environment Research for Temperate East Asia, Institute of Atmospheric Physics, Chinese Academy of Sciences, Beijing, China

**Keywords:** PM_10_/PM_2.5_, Chemical species, Light extinction, Potential contribution source function, Principal component analysis, Yulin

## Abstract

Daily PM_10_and PM_2.5_ sampling was conducted during four seasons from December 2013 to October 2014 at three monitoring sites over Yulin, a desert margin city. PM_10_ and PM_2.5_ levels, water soluble ions, organic carbon (OC), and elemental carbon (EC) were also analyzed to characterize their chemical profiles. *b*_*ext*_ (light extinction coefficient) was calculated, which showed the highest in winter with an average of 232.95 ± 154.88 Mm^−1^, followed by autumn, summer, spring. Light extinction source apportionment results investigated (NH_4_)_2_SO_4_ and NH_4_NO_3_ played key roles in the light extinction under high RH conditions during summer and winter. Sulfate, nitrate and Ca^2 +^ dominated in PM_10_/PM_2.5_ ions. Ion balance results illustrated that PM samples were alkaline, and PM_10_ samples were more alkaline than PM_2.5_. High SO_4_^2−^/K^+^ and Cl^−^/K^+^ ratio indicated the important contribution of coal combustion, which was consistent with the OC/EC regression equation intercepts results. Principal component analysis (PCA) analyses results showed that the fugitive dust was the most major source of PM, followed by coal combustion & gasoline vehicle emissions, secondary formation and diesel vehicle emissions. Potential contribution source function (PSCF) results suggested that local emissions, as well as certain regional transport from northwesterly and southerly areas contributed to PM_2.5_ loadings during the whole year. Local government should take some measures to reduce the PM levels.

## Introduction

Atmospheric aerosols have been found to be associated with adverse influences on atmospheric visibility, human health, and global climate change (Watson, 2002; Yuan et al., 2006; Cao et al., 2009; Huang et al., 2014). Aerosol extinction (scattering and absorption) plays the key role in the earth system (such as, radiative balance and energy budget) (Charlson et al., 1992). Chemical components of PM contributed to extinction can establish control measurement to alleviate visibility degradation (Tao et al., 2015). Sulfate, nitrate, organic matter (OM) and elemental carbon (EC) have been considered as dominant components of PM (Cao et al., 2003; Shen et al., 2014). All of the chemical compounds contributed to visibility degradation (Lee et al., 2009).

Yulin (36.95°−39.58°N, 107.46°−111.25°E), located in the Mu Us Desert, is one of the Asian Pacific Regional Aerosol Characterization Experiment (ACE-Asia) super site. However, most studies carried in Yulin have been studied to determine the chemical and physical profiles of Asian dust and its transportation (Xu et al., 2004). As the national energy and chemical industrial base, fossil fuel consumption and motor vehicles have rapidly increased in Yulin because of the economic growth, population expansion and urbanization. Actually, a study has been conducted recently aiming to understand brown carbon (BrC) can be also emitted from coal combustion in Yulin (Lei et al., 2018). However, seasonal PM levels, chemical species and visibility degradation are still lacking. It is not known dominant chemical components to light extinction during different seasons. Such information will offer practical and significant values in making relevant control to increase the atmospheric visibility. In this study, PM sampling was conducted in three represented sites for one year, and water-soluble ions and carbonaceous species (OC/EC) were also measured to understand PM pollution and their potential sources.

## Materials & Methods

### Sample collection

**Figure 1:**
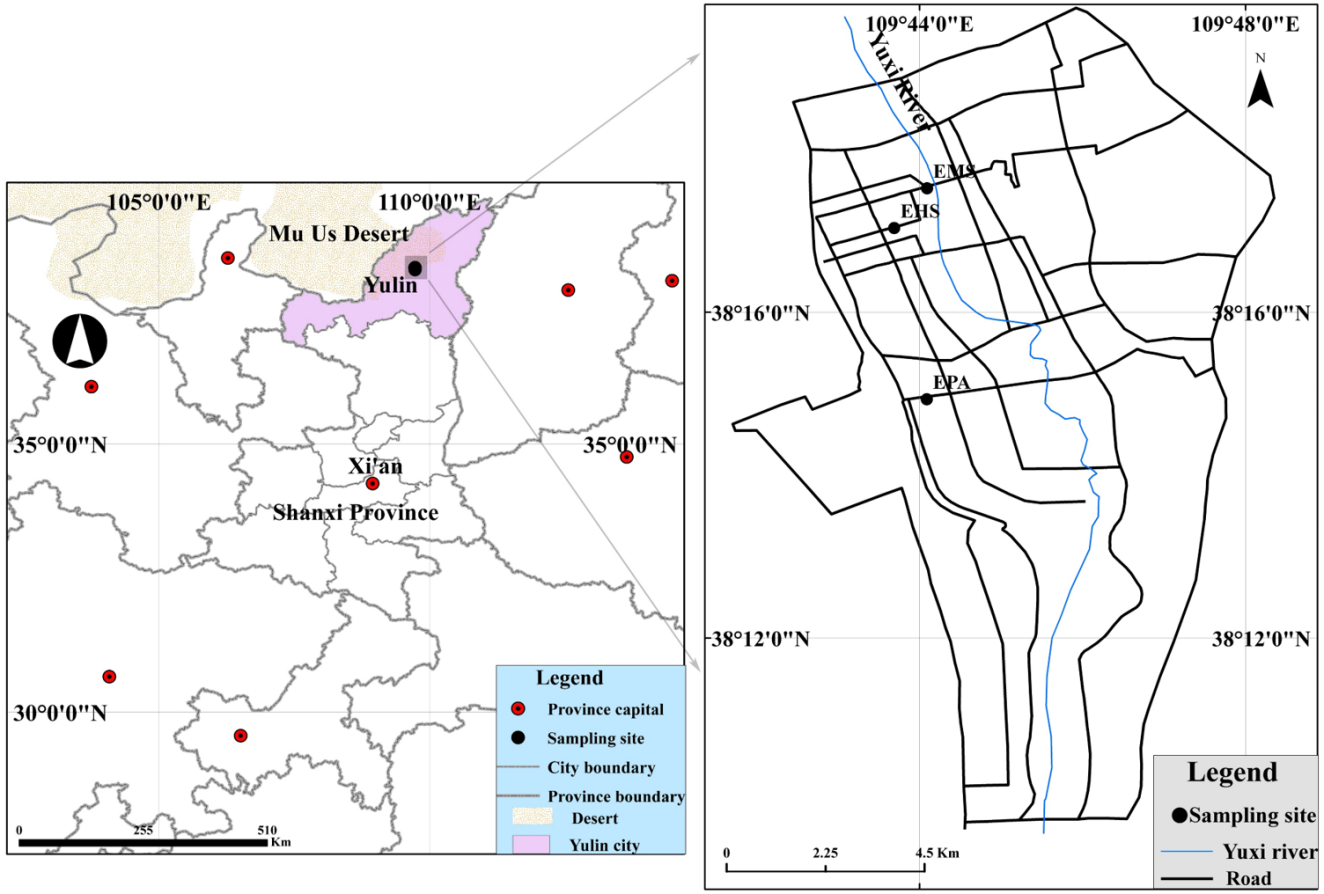
Locations of the monitoring sites and surrounding region.

Samplings were conducted at three sites (Fig. 1): Environmental Monitoring Station (EMS) is a mixed commercial-residential-traffic site; Experimental High School (EHS) represents a residential site, and Environmental Protection Agency (EPA) is considered as rural areas. All the sampling sites have been permitted and coordinated by the Yulin Environmental Monitoring Station. We selected four months for each season which were winter (December 2013), spring (April 2014), summer (July 2014), October (autumn 2014). PM samples were collected by six mini-volume samplers (Airmetrics, Springfield, OR) at 5 L min^−1^. Table S1 listed the sampling information about characteristics of the PM fraction sampling measurements. 121 PM_10_ and 285 PM_2.5_ samples were collected onto 47-mm quartz microfiber filters (Whatman, Maidstone, UK) using six minivolume samplers (Airmetrics, Springfield, OR) at 5 L/min. Before sampling, the quartz filters were pre-heated to 800 °C for 3 h to remove any residual carbon. After sampling, the filters were placed in clean plastic cassettes and stored in a refrigerator at ∼4 °C until in order to minimize the evaporation of volatile components. More details can be found in Lei et al. (2018).

### Mass and chemical analysis

PM samples were equilibrated using controlled temperature (20–23 °C) and relative humidity (35–45%) desiccators for 24 h before and after sampling, and their mass loadings were determined gravimetrically using a Sartorius MC5 electronic microbalance (±1 µg sensitivity; Sartorius, Göttingen, Germany). Each filter was weighed at least three times before and after sampling after 24 h equilibration period. The differences among the three repeated weightings typically were less than 10 µg for blanks and 15 µg for sample filters. 1/4 of each filter sample was extracted by 10 mL distilled-deionized water (18.2 M Ω) to analyze ions. Cations were detected by a CS12A column (Dionex Co., Sunnyvale, CA) and 20 mM methanesulfonate as an eluent. Anions were separated by an AS11-HC column (Dionex Co., Sunnyvale, CA) using 20 mM KOH as the eluent with detection limits less than 0.05 mg/L (Chow & Watson, 1999). Standard reference materials produced by the National Research Center for Certified Reference Materials, China, were analyzed for quality assurance purposes. Blank values were subtracted from sample concentrations. One sample in each group of ten samples was selected to analyze twice for quality control purposes. Additional details of ions analysis can be found in Shen et al. (2008). OC and EC were analyzed using DRI Model 2001 Thermal/Optical Carbon Analyzer (Atmoslytic Inc., Calabasas, CA, USA) based on the thermal/optical reflectance (TOR) method (Chow et al., 2007). The analyzer was calibrated with CH_4_ daily. One replicate analysis was performed for each of 10 samples.

### Data analysis

#### Neutralization factor

The neutralization factor (NF) can be used to describe the interaction between cations and anions (Shen et al., 2012). The NF of NH_4_^+^, Ca^2+^, Mg^2+^ have been calculated using the formula below

(1) \begin{eqnarray*}N{F}_{x}= \frac{X}{N{{O}_{3}}^{-}+S{{O}_{4}}^{2-}} \end{eqnarray*}

Where X may be NH_4_^+^, Ca^2+^ or Mg^2+^, using their equivalent concentrations (microgram per cubic meter).

#### Light extinction source apportionment

The light extinction coefficient (*b*_*ext*_), is calculated as the PM_2.5_ scattering (*b*_*sp*_, Chow et al., 2002), PM_2.5_ absorption (*b*_*ap*_, Chow et al., 2010), gas (NO_2_) absorption (*b*_*ag*_, Hodkinson, 1966), and Rayleigh scattering (*b*_*sg*_, Cao et al., 2012b), where:

(2) \begin{eqnarray*}& & {b}_{ext}={b}_{sp}+{b}_{ap}+{b}_{ag}+{b}_{sg}\end{eqnarray*}

(3) \begin{eqnarray*}& & {b}_{ap}=10\times [EC]\end{eqnarray*}

(4) \begin{eqnarray*}& & {b}_{ag}=0.33\times [N{O}_{2}]\end{eqnarray*}

(5) \begin{eqnarray*}& & {b}_{sp,wet}=f(RH)\times {b}_{sp,dry}\end{eqnarray*}

*b*_*ext*_ values can be approximately using the visual range (VR) (Koschmieder, 1924)

(6) \begin{eqnarray*}VR= \frac{3.912}{{b}_{ext}} \end{eqnarray*}

The revised IMPROVE formula was described as follow (Pitchford et al., 2007):

(7) \begin{eqnarray*}{b}_{ext} & = & 2.2\times {f}_{s}(\mathrm{RH})[(N{H}_{4})_{2}S{O}_{4small}]+4.8\times {f}_{s}(\mathrm{RH})[(N{H}_{4})_{2}S{O}_{4large}] & & +2.4\times {f}_{s}(\mathrm{RH})[N{H}_{4}N{O}_{3small}]+5.1\times {f}_{L}(\mathrm{RH})\times [N{H}_{4}N{O}_{3large}]+2.8\times [{\mathrm{OM}}_{small}] & & +6.1\times [{\mathrm{OM}}_{large}]+10\times [\mathrm{EC}]+1\times [\text{soil dust}]+1.7\times {f}_{ss}(\mathrm{RH})\times [\text{seasalt}] & & +0.6\times [\text{coarse mass}]+0.33\times [{\mathrm{NO}}_{2}]+\text{Rayleigh scattering}(\text{site specific})\end{eqnarray*}

The Large sulfate (*sulfate*_*large*_) and Small sulfate (*sulfate*_*small*_) are accumulated using the IMPROVE equation (John et al., 1990):

(8) \begin{eqnarray*}& & [(N{H}_{4})_{2}S{O}_{4large}]= \frac{[(N{H}_{4})_{2}S{O}_{4\mathrm{Total}}]^{2}}{20} \qquad \text{for} [(N{H}_{4})_{2}S{O}_{4\mathrm{Total}}]\lt 20 \mathrm{\mu }g{m}^{-3}\end{eqnarray*}

(9) \begin{eqnarray*}& & [(N{H}_{4})_{2}S{O}_{4large}]=[(N{H}_{4})_{2}S{O}_{4\mathrm{Total}}] \qquad \text{for} [(N{H}_{4})_{2}S{O}_{4\mathrm{Total}}]\geq 20 \mathrm{\mu }g{m}^{-3}\end{eqnarray*}

(10) \begin{eqnarray*}& & [(N{H}_{4})_{2}S{O}_{4small}]=[(N{H}_{4})_{2}S{O}_{4\mathrm{Total}}]-[(N{H}_{4})_{2}S{O}_{4large}]\end{eqnarray*}

The soil fraction was estimated as follows (Taylor & McLenna, 1985):

(11) \begin{eqnarray*}[\text{soil dust}]=( \frac{1}{0.035} )\times [Fe]=28.57\times [Fe]\end{eqnarray*}

#### Potential contribution source function method

Potential contribution source function (PSCF) is in view of backward trajectories which connects the residence time in upwind areas with relative high concentrations of a certain species through conditional probabilities (Polissar, Hopke & Harris, 2001). The PSCF method can be described as follows:

(12) \begin{eqnarray*}PSCF(i,j)={w}_{ij}\times ( \frac{{m}_{ij}}{{n}_{ij}} )\end{eqnarray*}

where w_ij_ is an arbitrary weight function to decrease small values effect of n_ij_. n_ij_ the number of the end points; m_ij_ is the number of trajectory end points in this grid cell whose values higher than the threshold value. In addition, the airmass backward trajectories had been previously calculated based on the National Oceanic and Atmospheric Administration (NOAA) Hybrid Single-Particle Lagrangian Integrated Trajectory model (Ji et al., 2019).

#### Principal component analysis (PCA) model

PCA is used to investigate the correlations among concentrations of chemical species at the receptor. The principal chemical species suggested the chemical species data variance and relevant possible sources (Viana et al., 2006; Meng et al., 2015). Each factor showed the maximum total variance of the data set and this set is completely uncorrelated with the rest of the data. The factor loadings obtained after the varimax rotation gives the correlation between the variables and the factor (Cao et al., 2005). SPSS™ and Statgraphics can be utilized to perform multivariate factor analysis. As elements are treated equally no matter their concentrations, original variables should be normalized as follows:

(13) \begin{eqnarray*}{Z}_{ij}= \frac{({\mathrm{x}}_{\mathrm{ij}}-{\overline{x}}_{i})}{{\sigma }_{i}} \end{eqnarray*}

Where *x*_*ij*_ is the *i* th mass concentration of each specie measured in the *j* th sample; *x*_*i*_ is the average *i* th element mass concentration and *σ*_*i*_ is the standard deviation.

For each source PCA identified, the weighted regression of each PM fraction’s concentration on the predicted PM contribution yields estimates of the content of that fraction in each source. More detailed introduction of this analysis method can be found in Cao et al. (2005).

#### Meteorological data

Meteorological data, including ambient temperature, relative humidity (RH), wind speed (WS), and atmospheric pressure, were collected from Weather Underground (http://www.wunderground.com/).

## Results

### Chemical composition in PM

Annual PM and chemical species in Yulin are summarized in Table 1. Generally, the yearly mean levels of PM_10_ and PM_2.5_ were 121.5 µg m^−3^ and 65.0 µg m^−3^. PM_10_ OC and EC levels were 17.9 µg m^−3^ and 5.5 µg m^−3^; for PM_2.5_, the values were 13.8 µg m^−3^ and 4.0 µg m^−3^. The total ions levels of PM_10_ and PM_2.5_ were 34.6 µg m^−3^ and 25.9 µg m^−3^, which accounted for 28.4% and 39.9% of the PM_10_ and PM_2.5_ mass, indicating that water-soluble ions comprise a large part of aerosol particles. The spatial distribution pattern showed that the PM_10_ and PM_2.5_ levels at EPA were the lowest compared with EMS and EHS. OC and EC levels showed a similar spatial distribution pattern as the PM levels, that’s EMS >EHS >EPA. In addition, the total ions levels at EMS (35.4 µg m^−3^ for PM_10_ and 26.1 µg m^−3^ for PM_2.5_) and EHS (35.7 µg m^−3^ for PM_10_ and 26.8 µg m^−3^ for PM_2.5_) were higher than those at EPA (32.1 µg m^−3^ for PM_10_ and 24.9 µg m^−3^ for PM_2.5_). Sulfate, the most abundance component in PM, followed by Ca^2+^ and NO_3_^−^.

**Table 1:** Mass concentrations of PM and chemical species (Unit: µg m^−3^).

PM Fraction			Mass	Na^+^	NH_4_^+^	K^+^	Mg^2+^	Ca^2+^	F^−^	Cl^−^	NO_3_^−^	SO_4_^2−^	OC	EC	OC/EC
EMS	PM_10_	mean	135.9	3.7	2.4	0.7	0.5	7.4	0.3	3.0	5.5	12.1	21.3	6.6	3.4
	(*n* = 40)	SD	70.2	1.2	2.0	0.4	0.4	3.9	0.2	3.1	3.2	5.5	12.5	3.7	1.2
	PM_2.5_	mean	74.8	3.3	2.4	0.6	0.3	3.4	0.3	2.3	4.0	9.5	14.9	4.4	3.7
	(*n* = 95)	SD	32.9	0.9	2.3	0.3	0.2	1.9	0.2	1.7	3.3	5.5	6.6	2.3	1.0
EHS	PM_10_	mean	120.2	3.8	2.8	0.8	0.5	5.8	0.3	2.8	6.2	12.7	17.9	5.4	3.6
	(*n* = 41)	SD	68.9	1.9	2.9	0.7	0.4	3.4	0.2	3.5	5.2	7.8	10.5	3.3	1.3
	PM_2.5_	mean	63.0	3.4	2.8	0.6	0.2	2.5	0.3	2.4	4.3	10.2	14.5	4.0	4.1
	(*n* = 98)	SD	26.2	1.0	2.4	0.3	0.2	1.3	0.2	2.3	3.5	5.8	7.8	2.6	1.4
EPA	PM_10_	mean	108.4	3.5	2.3	0.6	0.5	5.7	0.3	2.1	5.6	11.7	14.6	4.6	3.5
	(*n* = 40)	SD	64.4	1.2	1.9	0.3	0.3	3.2	0.3	2.2	3.3	6.1	6.9	2.4	1.6
	PM_2.5_	mean	57.0	3.3	2.5	0.6	0.2	2.6	0.3	1.8	4.1	9.6	11.9	3.5	3.8
	(*n* = 92)	SD	25.2	0.9	2.3	0.3	0.2	1.3	0.2	1.6	3.5	5.6	5.3	2.0	1.3
Average	PM_10_	mean	121.5	3.6	2.5	0.7	0.5	6.3	0.3	2.6	5.8	12.2	17.9	5.5	3.5
	(*n* = 121)	SD	68.3	1.5	2.3	0.5	0.4	3.5	0.2	3.0	4.0	6.5	10.5	3.3	1.4
	PM_2.5_	mean	65.0	3.3	2.6	0.6	0.2	2.8	0.3	2.2	4.1	9.8	13.8	4.0	3.9
	(*n* = 285)	SD	29.2	1.0	2.3	0.3	0.2	1.6	0.2	1.9	3.4	5.6	6.8	2.4	1.3


**Figure 2:**
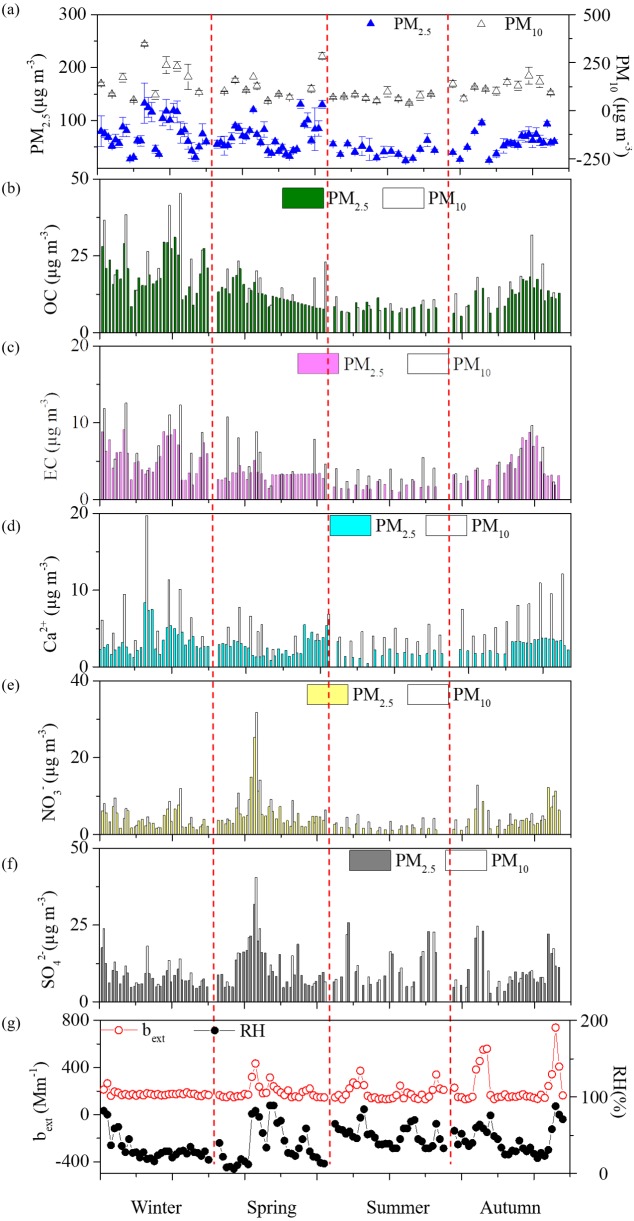
Temporal variations of (A) PM and their chemical components of (B) OC, (C) EC, (D) Ca^2+^, (E) NO_3_^−^, (F) SO_4_^2−^, and (G) bext and RH at Yulin during four seasons.

**Figure 3:**
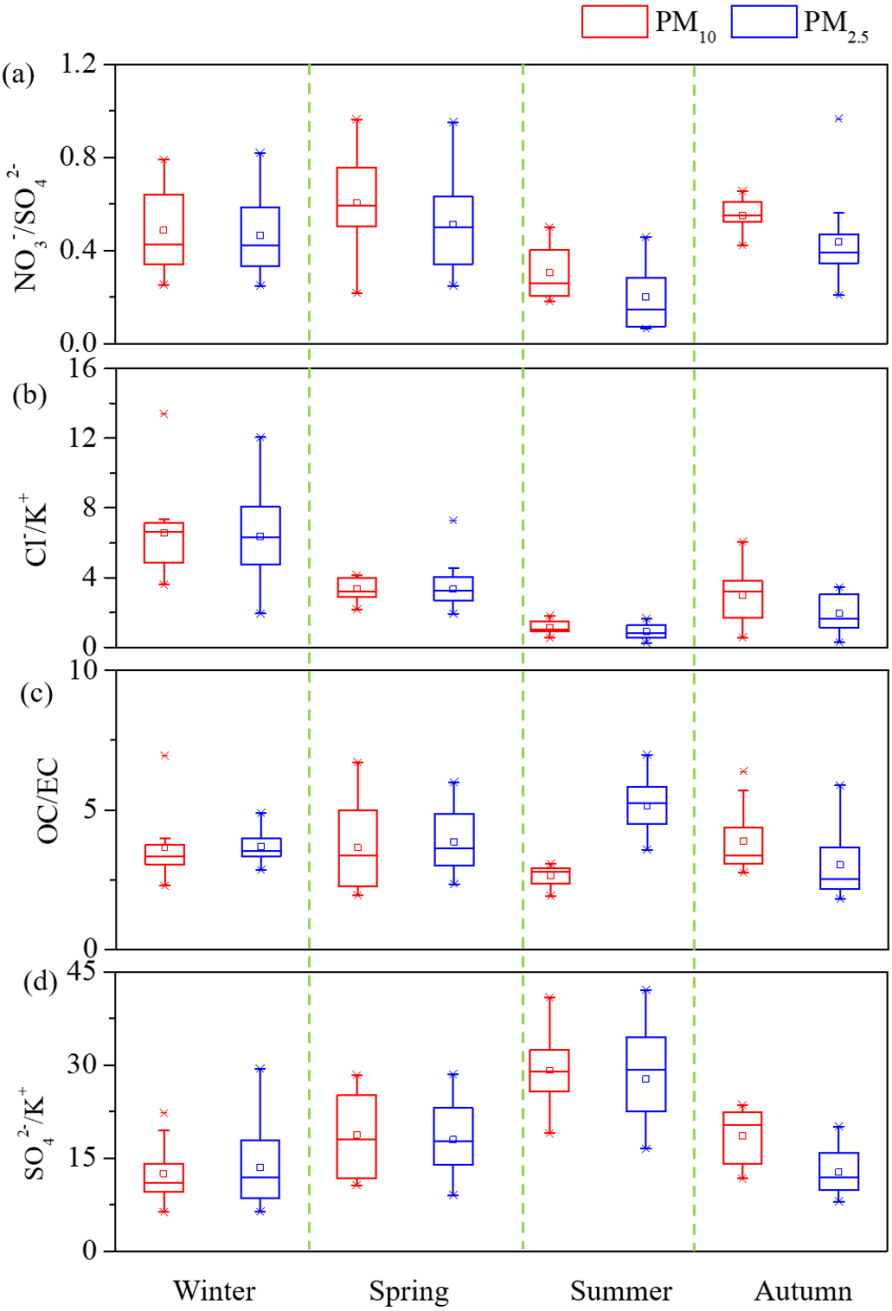
Box plot (10th, 25th, 50th, 75th, and 90th percentile; square pots: mean values) variations of (A) NO_3_^−^/SO_4_^2−^, (B) Cl^−^∕*K*^+^, (C) OC/EC and (D) SO_4_^2−^/K^+^ of PM.

Seasonal variations of PM, OC, EC, three major ions Ca^2+^, NO_3_^−^, and SO_4_^2−^ were shown in Fig. 2. In general, all the species were highest during winter but lowest during summer. During spring, PM_10_ and PM_2.5_ mean levels for dust dominated days were 283.4 µg m^−3^ and 130.4 µg m^−3^, which were both over 1.7 times of the spring average values. Two dust events were observed in winter and spring, leading the yearly highest PM_10_ Ca^2+^ concentrations of 19.7 µg m^−3^ (winter) and 12.9 µg m^−3^ (spring). The Ca^2+^ levels during dust events were over double of seasonal average values for both PM_10_ and PM_2.5_ and this phenomenon was consistent with the report of previous literatures (Wang et al., 2013; Shen et al., 2014). Box plot variations of NO_3_^−^/SO_4_^2−^, Cl^−^/K^+^, OC/EC, and SO_4_^2−^/K^+^ ratios have been also presented (Fig. 3) the average SO_4_^2−^/K^+^ ratios were 19.8 for PM_10_ and 18.0 for PM_2.5_. High SO_4_^2−^/K^+^ ratio indicated important coal combustion contribution to PM. In addition, Cl^−^/K^+^ ratios were 3.1.

The highest PM_2.5_ OC/EC ratios was observed during summer (5.2), followed by spring (3.9), winter (3.7), and autumn (3.0), which was consistent with the value as Cao et al. (2012a) illustrated. The regressions between OC and EC in PM_10_ and PM_2.5_ were plotted as shown, respectively (Fig. S1). Most spots of the PM_10_ and PM_2.5_ OC/EC ratios are displayed under the coal combustion line, which indicated important contribution of coal combustion (Cao et al., 2005). Strong correlation coefficients (R) of 0.87 for PM_10_ and 0.86 for PM_2.5_ were found between OC and EC. High correlations (Fig. S2) showed cations and anions were the major ions extracted from the PM samples. The seasonal A/C ratios were 0.8, 0.8, 0.6, and 0.7. The major fraction of deficit anions in spring should be carbonate concentration (CO_3_^2−^) (Shen et al., 2007; Shen et al., 2009). Material balance (Fig. S3) in the following parts revealed that mineral dust was one of major components in aerosol particle mass, and strong PM alkaline should attribute to high dust loading. A triangular diagram was created to show clearly the neutralization contribution of these three cations (Fig. S4). The yearly mean NF values of Ca^2+^, NH_4_^+^, and Mg^2+^ were 0.25, 0.17, and 0.02. It was clear that Ca^2+^ and NH_4_^+^ were the major neutralizers.

### Seasonal variations and source apportionment of light extinction

Daily averaged VR (Table S2) was 21.6 ± 7.3 km. Wind speeds (2.9 m s^−1^for both spring and summer) and temperature (13 °C for spring and 23 °C for summer) inferred higher mixing and dispersion than those during winter. As shown in Table 2, winter *b*_*ext*_ (calculated from section 2.3) showed the highest with an average of 232.95 ± 154.88 Mm^−1^, followed the decrease order of autumn >summer >spring, which were similar in Xi’an (Cao et al., 2012b).

**Table 2:** PCA performed on PM components, resulting in four independent factors.

Chemical species	PM_10_				PM_2.5_			
	Factor 1	Factor 2	Factor 3	Factor 4	Factor 1	Factor 2	Factor 3	Factor 4
Na^+^	0.655	−0.048	0.617	−0.335	0.627	−0.224	0.443	−0.285
NH_4_^+^	0.287	0.893	−0.145	0.226	0.201	0.951	0.107	−0.035
K^+^	0.69	0.386	0.318	−0.237	0.687	0.521	0.199	−0.185
Mg^2+^	0.55	−0.313	0.589	0.226	0.337	−0.416	0.693	−0.265
Ca^2+^	0.683	−0.37	0.414	0.186	0.408	−0.485	0.537	−0.221
F^−^	0.752	−0.024	−0.115	−0.384	0.665	−0.35	−0.118	−0.169
Cl^−^	0.813	0.008	0.262	−0.389	0.885	−0.117	−0.033	0.093
NO_3_^−^	0.374	0.776	0.209	0.174	0.343	0.735	0.372	−0.023
SO_4_^2−^	0.923	0.849	0.274	0.194	0.896	0.893	0.229	−0.096
OC1	0.773	0.07	−0.443	−0.238	0.898	−0.113	−0.196	−0.033
OC2	0.892	0.035	−0.33	0.058	0.908	0.091	−0.108	0.242
OC3	0.886	−0.123	−0.165	0.12	0.932	−0.058	−0.132	0.153
OC4	0.944	−0.221	−0.066	0.063	0.914	−0.097	−0.126	0.023
EC1	0.895	−0.014	−0.346	−0.024	0.912	0.057	−0.243	0.001
EC2	0.716	−0.114	−0.14	0.329	0.707	−0.212	0.081	0.366
EC3	0.531	−0.39	0.102	0.546	0.01	−0.017	0.653	0.712
OP	0.916	−0.003	−0.276	0.029	0.828	0.225	−0.314	−0.099
% Var	23.9	7.6	58.1	7.3	27.8	11.3	47.1	3.6

**Notes.**

% Var percentage of the variance explained by each factor

According to section 3.1 and mass balance results (Fig.S2), (NH_4_)_2_SO_4_, NH_4_NO_3_, OM, EC, the major contributors to PM. Moreover, coarse matter (CM) was important contributor to *b*_*ext*_ as a result of high PM_10_ concentrations (Cao et al., 2012a). In this study, unidentified chemical species were summed up and revered as “Others” below. In general, the *b*_*ext*_ can be estimated statistically as follows:

(14) \begin{eqnarray*}{b}_{ext} & = & {a}_{1}[\mathrm{CM}]+{a}_{2}[\mathrm{OM}]+{a}_{3}[\mathrm{EC}]+{a}_{4}{f}_{L}(RH)[{\mathrm{NH}}_{4}{\mathrm{NO}}_{3}]+{a}_{5}{f}_{L}(\mathrm{RH})[({\mathrm{NH}}_{4})_{2}{\mathrm{SO}}_{4}] & & +others\end{eqnarray*}

*b*_*ext*_ and chemical species mass concentrations are presented with the Mm^−1^ and µg m^−3^ unit, respectively. *f*_*L*_*(RH)* is the growth curves of sulfate and nitrate, which can be found in IMPROVE net results (Pitchford et al., 2007). *f*_*L*_*(RH)* was used in this study because sulfate and nitrate mass are distributed in droplet mode.

### Potential sources and transport pathways of PM_2.5_

**Figure 4:**
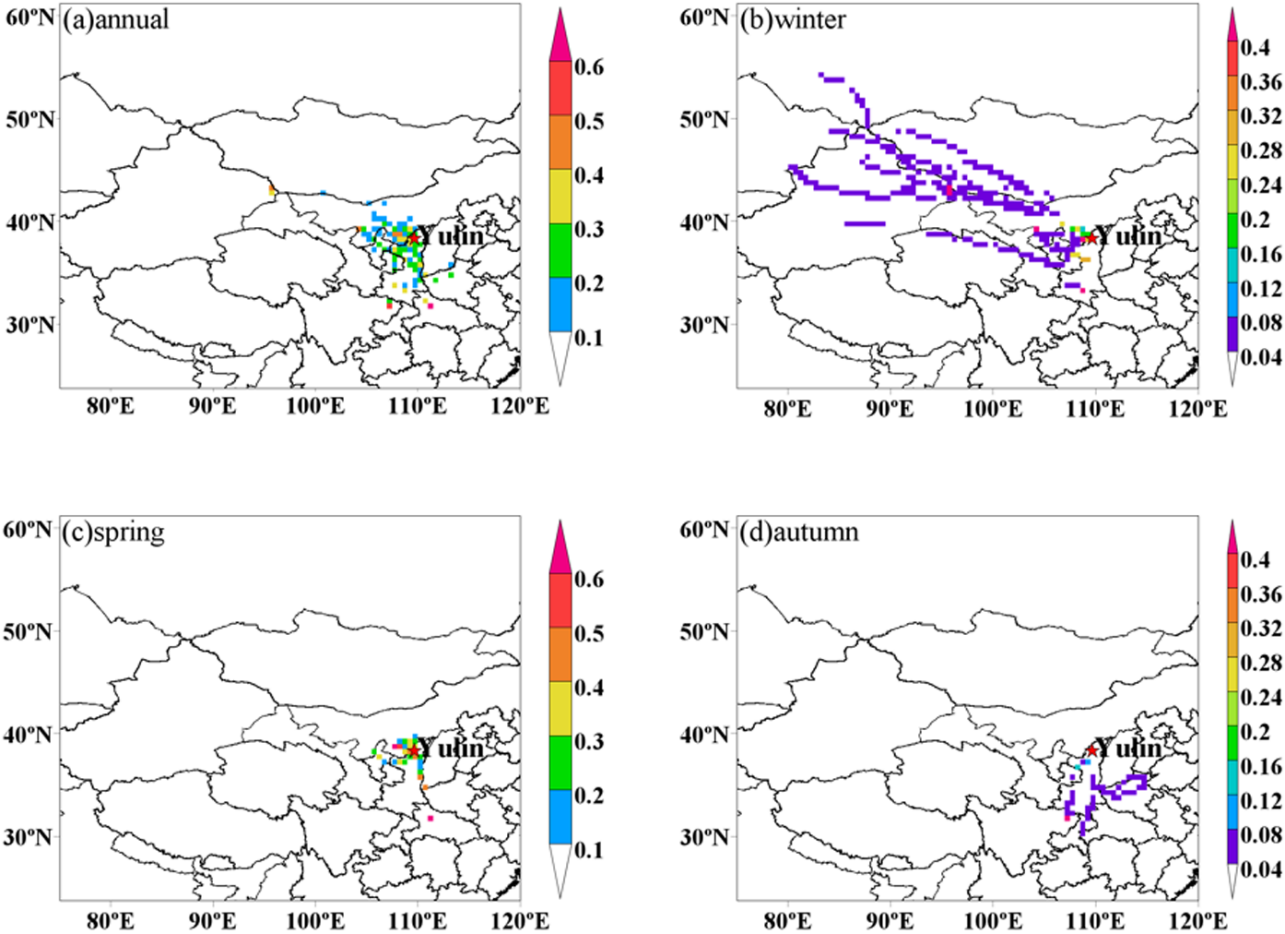
Potential source areas for PM_2.5_ in Yulin during (A) annual, (B) winter, (C) spring, and (D) autumn. The color code denotes the PSCF probability. The measurement site is indicated with a red circle.

In order to investigate the PM_2.5_ potential advection, PSCF analysis was conducted in this study (Petit et al., 2017). Figure 4 shows the PCSF analysis results from December 2013 to October 2014, which suggested that local emissions, as well as certain regional transport from northwesterly and southerly areas, contributed to PM_2.5_ loadings during the whole year. As average PM_2.5_ values were lower than 75 µg m^−3^ in summer, some differences were found during the other three seasons. During winter, the potential source area has been recorded; it was mainly from local emissions. Besides, low potential source area was from the northwestern plain areas of Ningxia Hui Autonomous Region and Xinjiang Uyghur Autonomous Region. In contrast to winter, higher potential source regions for PM_2.5_ during spring stretched to local emissions and the juncture of Guanzhong Plain, Henan province, Inner Mongolia and Ningxia Hui Autonomous Region.

### Source apportionment of PM

In this study, PCA analyses have been conducted to apportion the PM sources. The fundamental principle of PCA is that a strong correlation may exist between components from the same source. It searches factors that play the leading roles by analysis of correlation and variance. Multivariate factor analysis was adopted to help identification of dominant source categories and the results obtained by varimax rotated factor analysis for both PM_10_ and PM_2.5_ are presented in Table 3.

**Table 3:** PCA with varimax rotation for PM components data.

Chemical species	PM_10_					PM_2.5_				
	Factor 1	Factor 2	Factor 3	Factor 4	Communality	Factor 1	Factor 2	Factor 3	Factor 4	Communality
Na^+^	0.655	0.048	0.617	0.335	0.8234	0.627	0.224	0.443	0.285	0.8189
NH_4_^+^	0.287	**0.893**	0.145	0.226	0.8434	0.201	**0.951**	0.107	0.035	0.8674
K^+^	0.69	0.386	0.318	0.237	0.9123	0.687	0.521	0.199	0.185	0.9312
Mg^2+^	0.55	0.313	**0.589**	0.226	0.8822	0.337	0.416	**0.693**	0.265	0.8659
Ca^2+^	0.683	−0.37	**0.414**	0.186	0.8956	0.408	0.485	**0.537**	0.221	0.9187
F^−^	0.752	0.024	0.115	0.384	0.9231	0.665	−0.35	0.118	0.169	0.9052
Cl^−^	**0.813**	0.008	0.262	0.389	0.9453	**0.885**	0.117	0.033	0.093	0.9312
NO_3_^−^	0.374	**0.776**	0.209	0.174	0.9204	0.343	**0.735**	0.372	0.023	0.9124
SO_4_^2−^	**0.923**	**0.849**	0.274	0.194	0.9663	**0.896**	**0.893**	0.229	0.096	0.9645
OC1	**0.773**	0.07	0.443	0.238	0.8154	**0.898**	0.113	0.196	0.033	0.8069
OC2	**0.892**	0.035	−0.33	0.058	0.8798	**0.908**	0.091	0.108	0.242	0.8123
OC3	**0.886**	0.123	0.165	0.12	0.8332	**0.932**	0.058	0.132	0.153	0.8397
OC4	**0.944**	0.221	0.066	0.063	0.8120	**0.914**	0.097	0.126	0.023	0.8189
EC1	**0.895**	0.014	0.346	0.024	0.8987	**0.912**	0.057	0.243	0.001	0.8759
EC2	**0.716**	0.114	−0.14	0.329	0.8824	**0.707**	0.212	0.081	0.366	0.9325
EC3	0.531	−0.39	0.102	**0.546**	0.7923	0.01	0.017	0.653	**0.712**	0.8261
OP	**0.916**	0.003	0.276	0.029	0.8090	**0.828**	0.225	0.314	0.099	0.8267
% Var	23.9	7.6	58.1	7.3	Total 96.9%	27.8	11.3	47.1	3.6	Total 89.8%
Eigen value	5.34	3.45	2.67	1.6		6.52	2.87	2.91	1.23	

**Notes.**

% Var percentage of the variance explained by each factor

**Figure 5:**
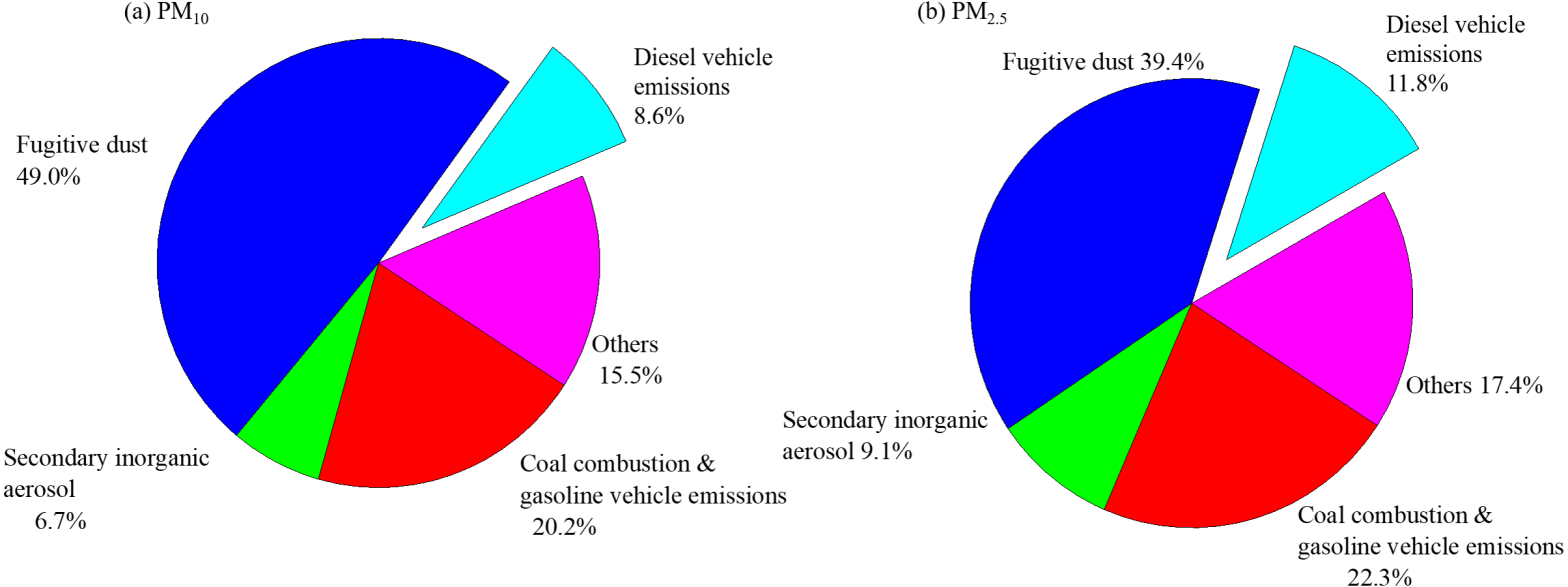
Source contribution analyses for (A) PM_10_ and (B) PM_2.5_ defined by PCA analyses.

The fugitive dust was the most major source of PM, followed by coal combustion & gasoline vehicle emissions, secondary inorganic aerosol, and diesel vehicle emissions for both PM_10_ and PM_2.5_ (Fig. 5). For PM_10_, Factor 1 was responsible for 23.9% of the total variance and had highly positive contributions from SO_4_^2−^, Cl^−^, OC four fractions, EC1, EC2, and OP, indicating its relation to coal combustion and gasoline vehicle emission (Huang et al., 2013). Factor 2 (7.6% of the total variance) had highly correlation with NH_4_^+^, SO_4_^2−^, and NO_3_^−^, which represented the source of secondary inorganic aerosols (Shen et al., 2011). The fugitive dust was a main contributor to PM_10_, with a contribution of 58.1% (Zhang et al., 2014). Factor 4 should be responsible for 7.3% of the total variance and had highly positive contributions from EC3, indicating its relation to diesel vehicle exhaust (Shen et al., 2011). However, PM_2.5_ showed some differences. Factors 1, 2, 3, and 4 represented the source of, diesel exhaust, coal combustion& gasoline exhaust, secondary inorganic aerosols, fugitive dust and diesel vehicle exhaust, accounting for 27.8%, 11.3%, 47.1% and 3.6% of the total variance, respectively.

The source apportionments of PM were presented in Fig. 5. PM_10_ showed a maximum contribution of 49.0% from fugitive dust. The coal combustion& gasoline vehicle emissions contributed 20.2%, while the contribution from secondary inorganic aerosols and diesel vehicle exhaust were found to be 6.7% and 8.6%, respectively. In the case of PM_2.5_, 39.4% of the mass has been contributed by 39.4% and 22.3% from coal combustion& gasoline vehicle emissions. Secondary inorganic aerosol contributed to 9.1% and 11.8% for diesel vehicle exhaust.

## Discussion

On a basis of a record of data analysis, this study has demonstrated that fossil fuel especially coal consumption should lead to the high PM pollutions in Yulin. In fact, in our previous study, lower NO_3_^−^/SO_4_^2−^ ratio suggests that stationary emission of coal combustion was the dominant source of PM particle (Lei et al., 2018). Coal burning in power plant and resident heating should be the major source of high sulfate. In addition, Industry emissions such as coke production also contributed to high sulfate. Sulfate, the most abundance component, highlighted coal combustion contribution to PM.

As Yulin is located in the cross border between desert and the Chinese loess, high spring PM levels should attribute to increasing of eolian dust. Prior studies also reported that heavy dust storm events in Yulin led to high TSP levels in spring of 257 µg m^−3^ (Zhang et al., 2003). In contrast, cities far away from the desert showed a different seasonal pattern compared to Yulin. High Ca^2+^ levels in winter and autumn should attribute to urban fugitive dust emitted by high wind from road and construction sites. The domestic heating period in Yulin started from November 1 to April 30 the next year. One hand, high SO_4_^2−^ concentrations observed during spring was mainly due to the domestic heat. The average wind speed and RH were 2.86 m s^−1^ and 38%, which were a little higher than those during summer. However, the temperature during spring was 13 °C, which could enhance the strong gas-particle transfer conversions of SO_2_ to SO_4_^2−^. In contrast, high summer sulfate should mainly due to high temperature enhancing the strong gas-particle transfer conversions of SO_2_ to SO_4_^2−^, as suggested in some studies (Wang et al., 2012; Shen et al., 2014). Winter lower SO_4_^2−^ levels attribute to high wind speed (2.56 m s^−1^ in average) favorable to diffusion and low relative humidity (33% in average) unfavorable to sulfate formation. High winter and low spring sulfate were observed in Xi’an, which showed a difference seasonal pattern when compared to Yulin (Shen et al., 2014). High winter sulfate was due to coal combustion in heating season and unfavorable diffusion condition in Xi’an (low wind speed, averaged 1.45 m s^−1^, and high RH, 55.9% in average). Lower NO_3_^−^/SO_4_^2−^ ratio suggests that stationary emissions are a dominant source of PM particles which has also been reported in Lei et al. (2018). Summer NO_3_^−^/SO_4_^2−^ ratios were the lowest for both PM_10_ and PM_2.5_, which because high temperature can favor SO_2_ converted to SO_4_^2−^, while low RH was unfavorable the NO_3_^−^ formation (Shen et al., 2008). High SO_4_^2−^/K^+^ and Cl^−^/K^+^ ratios indicated important coal combustion contribution to PM (Shen et al., 2009).

High summer PM_2.5_ OC/EC ratio inferred a dominant fraction of OC from gas-particle conversion. In fact, high temperature and atmospheric oxidation in summer favored the secondary organic carbon (SOC) formation (Wang et al., 2012). The regressions between OC and EC in PM_10_ and PM_2.5_ were plotted as shown, respectively (Fig. S1). Most spots of the PM_10_ and PM_2.5_ OC/EC ratios are displayed under the coal combustion line, which indicated important contribution of coal combustion (Cao et al., 2005). High sulfate and OC levels also supported our conclusion. The regression equation intercepts for PM_2.5_ and PM_10_, indicated that OC primary non-combustion emissions, such as regional background carbonaceous species, long range transport, and local biological detritus, influenced heavily on fine particles in comparison with coarse fraction (Turpin & Huntzicker, 1995). Seasonal A/C ratios variations showed that spring PM samples were more alkaline because of the high loadings of Ca^2+^ and Mg^2+^ (Shen et al., 2014). The neutralization contributions illustrated that low contribution of Mg^2+^ changed the PM from weakly acidic to weakly alkaline in many PM samples.

Good correlations in different seasons were found between the reconstructed b_ext_ and the *b*_*ext*_ estimated by visibility (Fig. S5). A summary of the light extinction source apportionment results were presented in Table 2. On average, CM, NH_4_NO_3_ and(NH_4_)_2_SO_4_ were the most chemical species contributing to *b*_*ext*_. (NH_4_)_2_SO_4_ accounted highest in summer (34.39 ± 14.79%), while NH_4_NO_3_showed the large contribution in autumn. During winter, (NH_4_)_2_SO_4_ also was the highest during winter followed by CM. These results indicated that the extinction effects from (NH_4_)_2_SO_4_ and NH_4_NO_3_ significantly increased under high RH conditions during summer and winter.

PSCF results have shown that dust was the most abundant components during spring, which was due to the atmospheric dust transport. Unlike winter and spring, potential source area was mainly from local emissions and low potential source areas were from northerly areas like Guanzhong Plain. This is consistent with the dominant source from coal combustion related above (Liu et al., 2015). PCA results showed the fugitive dust and coal combustion dominated the PM loadings both in PM_10_ and PM_2.5_ over Yulin. Moreover, local emissions and some certain regional transport were the main sources. Despite the dust transportation, the economic boom in Yulin gave rise to substantial air pollution. In order to improve the air quality, strict measurements should be launched in both local and regional areas.

## Conclusions

The chemical species for PM were analyzed and their associated sources were identified in Yulin, China. PM levels, OC, EC were highest during winter and lowest during summer. High Ca^2+^ levels during winter and autumn should attribute to fugitive dust. High spring and summer sulfate levels should be due to different sources. Ion balance illustrated that PM_10_ samples were more alkaline than PM_2.5_. Winter *b*_*ext*_ showed the highest with an average of 232.95 ± 154.88 Mm^−1^, followed by autumn, summer, and spring. The regression equation intercepts for different values in PM_2.5_ and PM_10_ indicated that OC primary non-combustion emissions, such as regional background carbonaceous species, long range transport, and local biological detritus, influenced heavily on fine particles in comparison with coarse fraction. Light extinction source apportionment results inferred that the extinction effects from hygroscopic species, such as, (NH_4_)_2_SO_4_, NH_4_NO_3_, increased significantly under high RH conditions during summer and winter. High SO_4_^2−^/K^+^ and Cl^−^/K^+^ ratio indicated the important contribution of coal combustion. PCA analyses results showed that the fugitive dust was the most major source of PM, followed by coal combustion & gasoline vehicle emissions, secondary inorganic aerosol and diesel vehicle emissions. PSCF results suggested that PM_2.5_ were mainly from both local emissions and regional transport from northwesterly and southerly areas during the whole year.

##  Supplemental Information

10.7717/peerj.8447/supp-1 Figure S1 OC-to-EC ratios in PM_10_ and PM_2.5_

10.7717/peerj.8447/supp-2 Figure S2 Total anion microequivalents versus total cation microequivalents: (a) the whole year for PM_10_, (b) stratified by seasons for PM_10_, (c) the whole year for PM_2.5_, (d) stratified by seasons for PM_2.5_

10.7717/peerj.8447/supp-3 Figure S3 Mass balance of chemical species in (a) PM_10_ and (b) PM_2.5_ for the year

10.7717/peerj.8447/supp-4 Figure S4 Triangular diagrams of NF for Mg^2+^, Ca^2+^, and NH_4_^+^ in (a) PM_10_ and (b) PM_2.5_

10.7717/peerj.8447/supp-5 Figure S5 Correlations between the reconstructed and the estimated b_*ext*_ in different seasons

10.7717/peerj.8447/supp-6 Table S1 Characteristics of the PM fraction sampling measurements (in sampling period, duration, and frequency)

10.7717/peerj.8447/supp-7 Table S2 Meteorological data during the sampling periods

10.7717/peerj.8447/supp-8 Data S1 Raw data

10.7717/peerj.8447/supp-9 Supplemental Information 9 Highlights

## Additional Information and Declarations

### Field Study Permissions

The following information was supplied relating to field study approvals (i.e., approving body and any reference numbers): All the field work have been permitted by the Yulin Environmental Monitoring Station (20150915).

### Data Availability

The following information was supplied regarding data availability: The raw measurements are available in the Supplemental Files.
