# Supplementary figures and images for: Aerosols chemical composition, light extinction, and source apportionment near a desert margin city, Yulin, China
Source: PeerJ. 2020 Feb 14;8:e8447. doi: 10.7717/peerj.8447 (PMC7025702; doi:10.7717/peerj.8447)

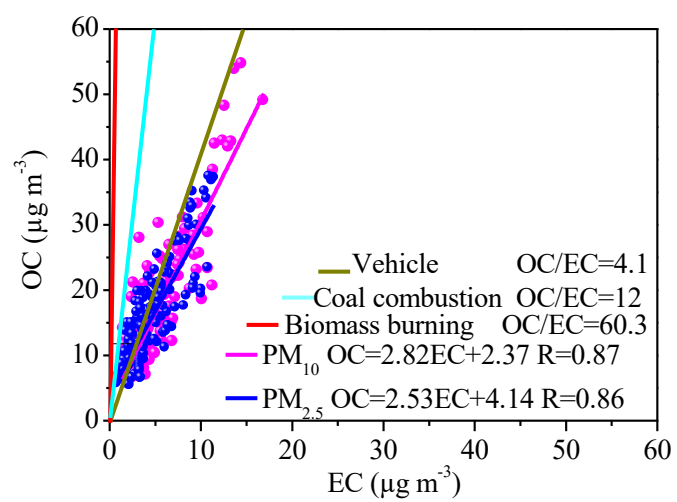

Supplement: Figure S1 [file peerj-08-8447-s001.pdf]

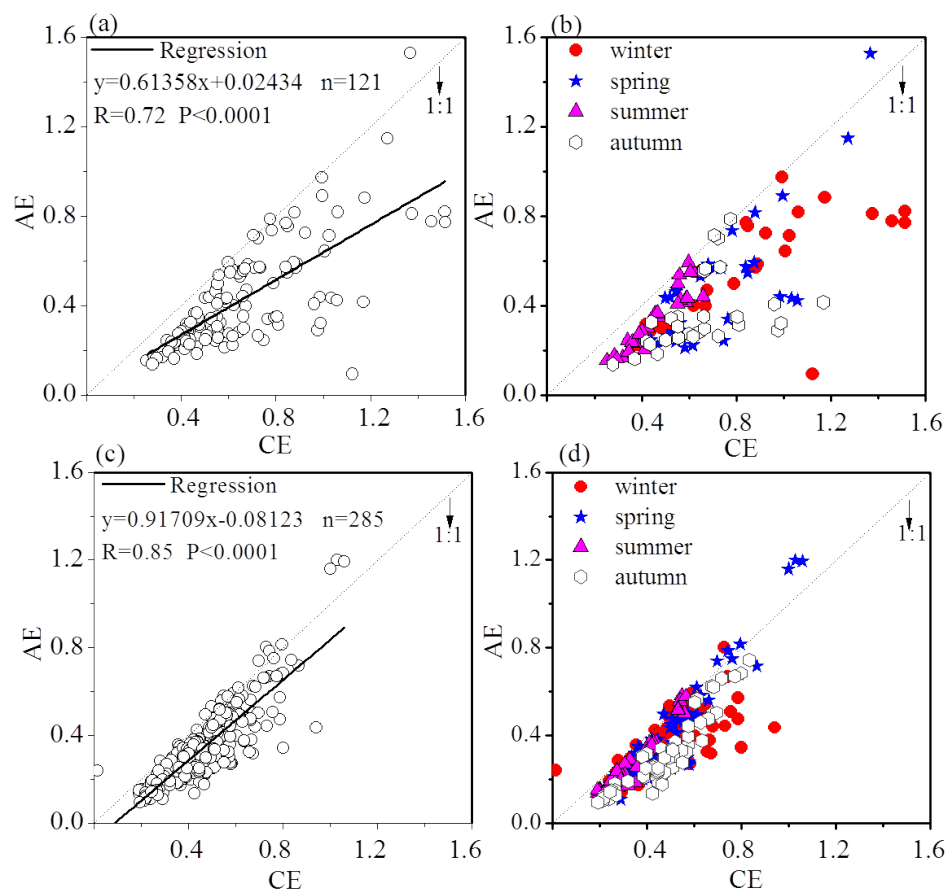

Supplement: Figure S2 [file peerj-08-8447-s002.pdf]

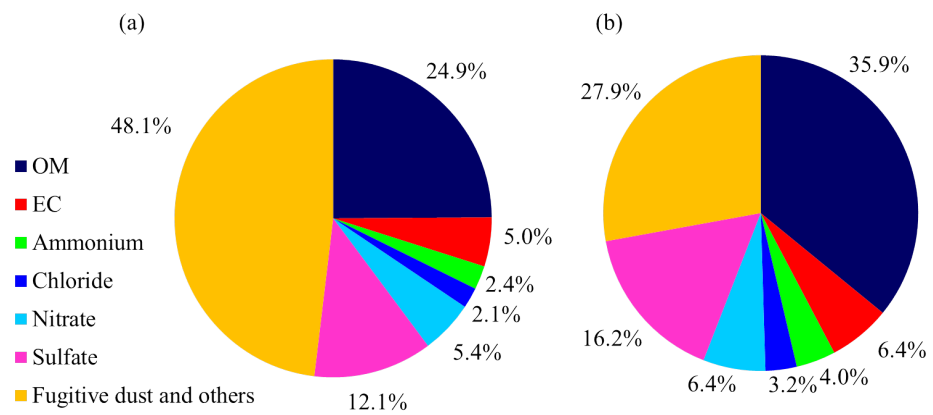

Supplement: Figure S3 [file peerj-08-8447-s003.pdf]

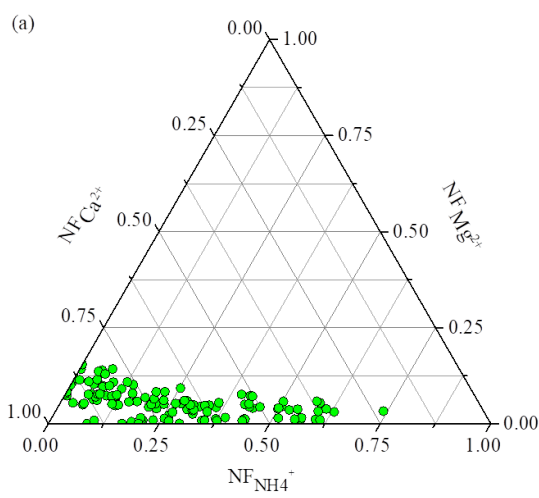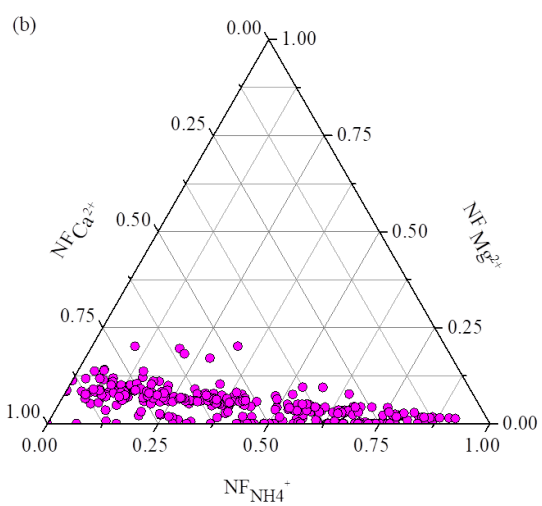

Supplement: Figure S4 [file peerj-08-8447-s004.pdf]

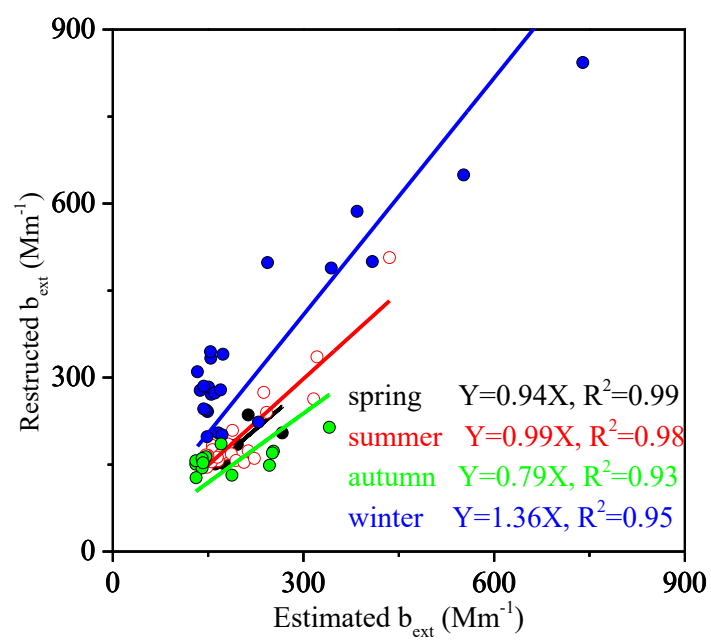

Supplement: Figure S5 [file peerj-08-8447-s005.pdf]
