# Supplementary material for: Aerosols chemical composition, light extinction, and source apportionment near a desert margin city, Yulin, China
Source: PeerJ. 2020 Feb 14;8:e8447. doi: 10.7717/peerj.8447 (PMC7025702; doi:10.7717/peerj.8447)
Supplement: Table S1 [file peerj-08-8447-s006.docx]

Table S1. Characteristics of the PM fraction sampling measurements (in sampling period, duration, and frequency)

| PM fraction | Sampling period | Sampling duration |
| --- | --- | --- |
| PM_10_ | April, 2014 | 24h (9:00a.m. to 9:00 a.m.) |
|  | July, 2014 | 48h (9:00a.m. to 9:00 a.m.) |
|  | October, 2014 | 48h (Oct. 1 to 13),  24h (Oct. 13 to 30)  (9:00a.m. to 9:00 a.m.) |
|  | December, 2013 | 24h (9:00a.m. to 9:00 a.m.) |
| PM_2.5_ | April, 2014 | 24h (9:00a.m. to 9:00 a.m.) |
|  | July, 2014 | 48h (9:00a.m. to 9:00 a.m.) |
|  | October, 2014 | 48h (October 1 to 13),  24h (October 13 to 30)  (9:00a.m. to 9:00 a.m.) |
|  | December, 2013 | 24h (9:00a.m. to 9:00 a.m.) |
