# Supplementary material for: Aerosols chemical composition, light extinction, and source apportionment near a desert margin city, Yulin, China
Source: PeerJ. 2020 Feb 14;8:e8447. doi: 10.7717/peerj.8447 (PMC7025702; doi:10.7717/peerj.8447)
Supplement: Table S2 [file peerj-08-8447-s007.docx]

Table S2 Meteorological data during the sampling periods

|  | Temperature(°C) | RH(%) | Wind speed(m/s) | VR(km) |
| --- | --- | --- | --- | --- |
| winter | 2.1 | 33.0 | 2.56 | 21.4 |
| spring | 13.0 | 38.0 | 2.86 | 21.6 |
| summer | 23.0 | 50.0 | 2.89 | 23.1 |
| autumn | 12.0 | 44.0 | 2.67 | 22.3 |
