# Supplementary material for: Aerosols chemical composition, light extinction, and source apportionment near a desert margin city, Yulin, China
Source: PeerJ. 2020 Feb 14;8:e8447. doi: 10.7717/peerj.8447 (PMC7025702; doi:10.7717/peerj.8447)
Supplement: Supplemental Information 9 [file peerj-08-8447-s009.docx]

Highlights

- Daily size-resolved PM samples were collected during four seasons over a desert city.
- High Ca^2+^ levels in winter and autumn should attribute to fugitive dust.
- Extinction effects of (NH_4_)_2_SO_4_ and NH_4_NO_3_ were significantly enhanced under high RH conditions during summer and winter.
- Fugitive dust was the most major source of PM over Yulin.
